# Protecting-Group-Free Synthesis of 2-Deoxy-Aza-Sugars

**DOI:** 10.3390/molecules14125298

**Published:** 2009-12-16

**Authors:** Emma Marie Dangerfield, Catherine Heather Plunkett, Bridget Louise Stocker, Mattie Simon Maria Timmer

**Affiliations:** 1 Malaghan Institute of Medical Research, P.O. Box 7060, Wellington, New Zealand; 2 School of Chemical and Physical Sciences, Victoria University of Wellington, P.O. Box 600, Wellington, New Zealand

**Keywords:** aza-sugar, glycosidase inhibitors, asymmetric, reductive amination, carbamate

## Abstract

The protecting-group-free asymmetric synthesis of 1,2,4-trideoxy-1,4-imino-L-xylitol is readily achieved in five steps from 2-deoxy-D-ribose and with an overall yield of 48%. Key in this synthesis is the application of our recently developed Vasella-reductive amination and carbamate annulation methodologies to the synthesis of 2-deoxy-aza-sugars. The carbamate annulation occurred with excellent yield and diastereoselectively (>20:1 *d.r.*), in favour of the 3,4-*cis* isomer.

## Introduction

Aza-sugars (also known as imino-sugars) are mimetics of native sugars whereby the ring oxygen is replaced by a nitrogen atom. Due to their structural similarity to native carbohydrates, aza-sugars are able to act as glycosidase inhibitors and therefore have enormous therapeutic potential in the treatment of a variety of diseases including viral infection, bacterial infection, lysosomal storage disorders, cancer and diabetes [1,2,3,4,5,6,7,8,9]. Indeed, the use of Zavesca^® ^for the treatment of Gaucher’s disease marked the launch of the first aza-sugar medication [10].

**Figure 1:**
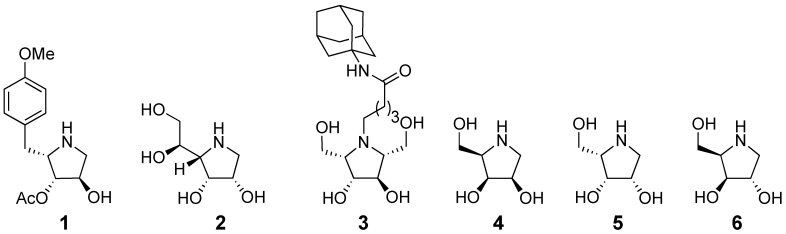
Representative pyrrolidines containing the 3,4-*cis* substituted core.

In view of the growing biological importance of aza-sugars, our interest has been in the development of new methodologies for the diastereoselective synthesis of this class of compounds [11]. In particular, our focus has been on the synthesis of 3,4-*cis*-substituted aza-sugars (Figure 1), which include family members such as the antibiotic Anisomycin (**1**) [12], the potent mannosidase inhibitor 1,4-dideoxy-1,4-imino-D-mannitol (**2**) [13], adamantyl substituted 2,5-anhydro-2,5-imino-D-glucitol **3**, a promising molecular chaperone in the treatment of Gaucher’s disease [14], the α-galactosidase inhibitor 1,4-dideoxy-1,4-imino-D-lyxitol (**4**) [15] and its L-isomer, 1,4-dideoxy-1,4-imino-L-lyxitol (**5**) [16]. Even in recent years, novel pyrrolidines, such as 1,4-dideoxy-1,4-imino-D-xylitol (**6**), are still being isolated from natural products [17].

Due to the limited biological availability of many aza-sugars, only few have been widely studied for their therapeutic potential. The ability to rapidly and efficiently synthesise aza-sugars therefore represents an enormous potential for full assessment of their biological profiles. The methodology we developed for the synthesis of 3,4-*cis*-substituted aza-sugars involves a protecting-group-free strategy whereby readily available carbohydrate precursors are transformed into 3,4-*cis*-substituted aza-sugars via use of a Vasella-reductive amination protocol and a novel carbamate annulation reaction [11]. Our strategy is not merely competitive in yield and number of linear-steps, but also employs many of the principles of Green Chemistry (*i.e.*, atom economy, reactions in aqueous media), and has been applied to the synthesis of 1,4-dideoxy-1,4-imino-L-lyxitol (**5**) and 1,4-dideoxy-1,4-imino-D-xylitol (**6**), each synthesised in five steps and in 55% and 57% overall yield, respectively. We now wish to report on the extension of our methodology for the synthesis of 2-deoxy-aza-sugars and to provide further explanation for the remarkable diastereoselectivity observed in our carbamate annulation reaction.

## Results and Discussion

**Scheme 1:**
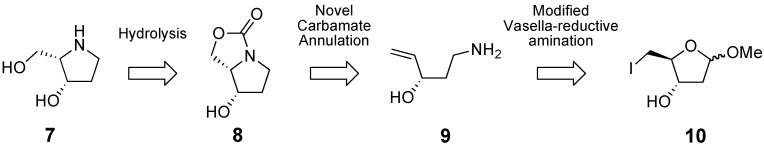
Retrosynthesis for the formation of 1,2,4-trideoxy-1,4-imino-L-xylitol.

The retrosynthesis of our target aza-sugar, 1,2,4-trideoxy-1,4-imino-L-xylitol (**7**), is presented in Scheme 1. Here, the pyrrolidine **7** is prepared via the hydrolysis of carbamate **8**, which is itself formed via an iodine-promoted halocyclization/*in situ* carbonylation reaction. The linear amino alcohol **9** is in turn accessible via the reductive amination of hydroxyaldehydes obtained following Vasella reaction of methyl iodofuranosides **10**, prepared in two steps from 2-deoxy-D-ribose. As can be seen, this synthetic route is short and efficient, and can be accomplished without the use of protecting groups. It is anticipated that the C-3 stereogenic centre in 2-deoxy-D-ribose will be key in determining the stereochemistry of carbamate **8**, and hence the final pyrrolidine **7**.

**Scheme 2:**
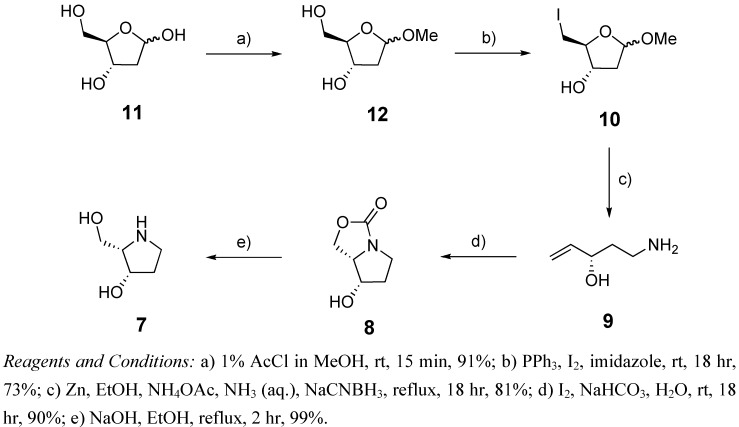
Synthesis of 1,2,4-trideoxy-1,4-imino-L-xylitol.

With our synthetic strategy in place, synthesis of 2-deoxy-aza-L-xylitol commenced with the formation of methyl glycoside **12** from 2-deoxy-D-ribose (**11**) under standard Fischer glycosidation conditions (Scheme 2). Given the propensity of 2-deoxy-D-ribose to form the thermodynamically more stable pyranose ring, short reaction times were required to favour formation of the desired furanoside. Thus, in accordance with a procedure by Hoffer and co-workers [18], 2-deoxy-D-ribose was subjected to 1% AcCl in MeOH for 15 min, resulting in almost exclusive formation of 2-deoxy-D-ribofuranoside **12** with only trace amounts (*ca.* 5%) of the pyranose structural isomer. Installation of an iodide at the primary position was then attempted by refluxing a solution of methyl 2-deoxy-D-ribose **12**, triphenylphosphine, I_2_ and imidazole in THF for 2 hr. Unfortunately, these conditions led to significant degradation, however milder reaction conditions, involving stirring the same reagents at room temperature for 18 hr [19], allowed for the preparation of methyl 2,5-dideoxy-5-iodo-D-riboside (**10**) in satisfactory yield (73%).

With the key methyl iodoglycoside precursor **10** in hand, we were then able to investigate the applicability of our Vasella-reductive amination conditions to the synthesis of linear alkenylamines without a substituent at the C-2 position. Previously we reported that primary amines containing hydroxy groups at C-2 and C-3 can be prepared in one step from the corresponding methyl iodo-glycoside [3]. Key in this reaction was the use of saturated NH_4_OAc in buffered conditions for the Vasella-reductive amination. Such conditions allow for the preferential formation of the desired primary amine over the formation of the undesired secondary amine, which can occur if the primary amine undergoes a second reductive amination reaction with another molecule of aldehyde (formed in the Vasella reductive ring opening). With an interest in exploring the scope of this reaction, we subjected methyl iodo-glycoside **10** to a suspension of Zn, NH_4_OAc, NH_3_ and NaCNBH_3_ in ethanol at reflux (Scheme 2). Upon completion of the reaction, as indicated by TLC, the reaction was concentrated and acidified, using isopropanol and hydrochloric acid, and the residue purified using a Dowex (H^+^) column, according to our previously published protocols [3]. These conditions led to the isolation of the desired alkenylamine **9** in *ca.* 70% yield, though the sample was contaminated with an undesired by-product, 1,5-diaminopent-2-ene (*ca.* 10%) as evidenced by a mass of 101.1082 [M+H^+^], the presence of an internal olefin (^13^C NMR δ 132.4 and δ 124.8) and HMBC between C3 and H2, H1 and H5, C4 and H2 and H5, C1 and H2, and C2 and H1, and with no HMBC between C5 and H2 or H1. It is proposed that this by-product was formed via displacement of the allylic hydroxyl of **9** following protonation upon work-up and nucleophilic attack by ammonia at the allylic position. Accordingly, a milder work-up procedure was performed, involving co-evaporation of the reaction mixture *in vacuo* with isopropanol, to remove the majority of the ammonium acetate salts, loading of the residue directly onto a Dowex (H^+^) column and elution with aqueous ammonia. Using this protocol, linear alkenylamine **9** was isolated in good (81%) yield and with no trace of the diamino by-product. Having illustrated that our modified Vasella-reductive amination conditions are also applicable to the synthesis of C-2 unsubstituted alkenylamines, alkenylamine **9** was then subjected to an iodine-promoted carbamate annulation (Scheme 2). To our delight, this reaction proceeded smoothly and gave the desired carbamate **8** in quantitative yield and with a diastereoselectivity of greater than 20:1 in favour of the 3,4-*cis* isomer, as determined by ^1^H NMR of the crude reaction mixture. The hydrolysis of carbamate **8** using NaOH proceeded uneventfully and quantitatively gave the desired pyrrolidine **7**, as confirmed via comparison of spectral data with that previously reported [20,21,22,23]. In sum, we have been able to prepare 1,2,4-trideoxy-1,4-imino-L-xylitol in 5 steps, from cheap and readily available 2-deoxy-ribose, in an overall yield of 48%, the shortest and highest yielding route reported to date.

Perhaps one of the most remarkable features of our strategy for the synthesis of pyrrolidines is the high yield and diastereoselectivity observed in the carbamate annulation reaction. The diastereoselectivity is in favour of the 3,4-*cis* isomer, as illustrated by formation of 2-deoxy L-*xylo* carbamate **8 **(entry 1, Table 1), L-*lyxo* carbamate **14 **(entry 2) and D-*xylo* carbamate **16 **(entry 3) [11] from alkenylamines **7**, **13**, and **15**, respectively. From these results it is apparent that the stereochemistry at the C-3 position exerts stereocontrol on the cyclisation reaction and is independent of the stereochemistry, or lack of stereochemistry, at the C-2 position.

**Table 1:** Diastereoselectivity of iodine-promoted carbamate annulations.

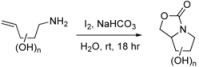				
Entry	Alkenylamine	Carbamate	Diastereoselectivity	Yield^a^
1	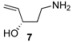	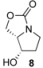	> 20:1	95%
2^b^	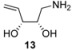	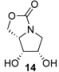	> 20:1	93%
3^b^	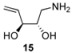	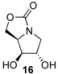	> 20:1	99%

^a^ Isolated yield. ^b ^See reference [11].

**Figure 2:**
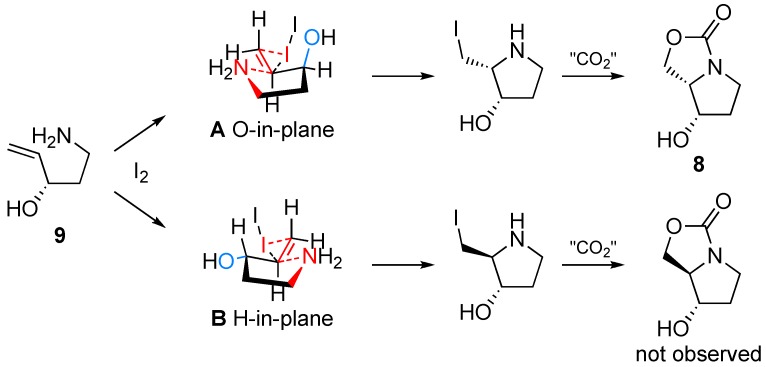
Proposed transition state for iodine-promoted carbamate annulation.

The high diastereoselectivity observed in these cyclisation reactions can be explained via a transition state model originally proposed by Chamberlin *et al.* [24,25,26] and in line with theoretical studies by Gouverneur and co-workers [27]. Attack of the amine on the I_2_–ethylene complex is envisioned to take place via a 5-membered ring transition structure, in which the ring nitrogen approaches the double bond in an ^N^*E* (envelope) conformation, and follows a Bürgi-Dunitz-like trajectory [28] (Figure 2). The hydroxyl substituent on the ring can now be positioned either in the plane of the double bond (**A**, O-in-plane), or almost perpendicular to that plane (**B**, H-in-plane). Of these two transition states, **A** has minimal overlap between the electron-withdrawing 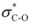 and reacting *π*_C=C_ orbitals, thereby forming the lowest energy transition state. The H-in-plane structure (**B**) has overlapping hydroxyl 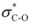 and double bond *π*_C=C_ orbitals, which destabilises the I_2_–π complex and is hence disfavoured.

## Experimental

### General procedures

Unless otherwise stated all reactions were performed under atmospheric air. THF (Lab-Scan) was distilled from LiAlH_4_ prior to use. H_2_O, MeOH (Pure Science), EtOAc (Pure Science), and Hexanes (Pure Science) were distilled prior to use. EtOH (absolute, Pure Science), AcOH (Ajax Finechem), DCM (LabServ), 30% aqueous NH_3_ (J. T. Baker Chemical Co.), Et_2_O (Merck), 2-deoxy-D-ribose (Fluka), AcCl (B&M), PPh_3_ (Merck), Imidazole (Aldrich), I_2_ (BDH), NaCNBH_3_ (Aldrich), NaOH (Pure Science), and NH_4_OAc (AnalaR) were used as received. Zn dust was activated by the careful addition of conc. H_2_SO_4_ washed with EtOH (3x) and hexanes (3x), and stored under dry hexanes. All solvents were removed by evaporation under reduced pressure. Reactions were monitored by TLC-analysis on Macherey-Nagel silica gel coated plastic sheets (0.20 mm, Polygram SIL G/UV_254_) with detection by UV-absorption (254 nm), by spraying with 20% H_2_SO_4_ in EtOH followed by charring at ~150 °C, by dipping in I_2_ in silica, or by spraying with a solution of ninhydrin in EtOH followed by charring at ~150 °C. Column chromatography was performed on Pure Science silica gel (40-63 micron). Dowex^®^ W50-X8 acidic resin (Sigma) and Dowex^®^ 1X4-50 basic resin (Sigma) were used for ion exchange chromatography and HP20 (Supelco) for reverse phase chromatography. High-resolution mass spectra were recorded on a Waters Q-TOF Premier^TM^ Tandem Mass Spectrometer using positive electro-spray ionisation. Optical rotations were recorded using a Perkin-Elmer 241 polarimeter at the sodium D-line. Infrared spectra were recorded as thin films using a Bruker Tensor 27 FTIR spectrometer, equipped with an Attenuated Total Reflectance (ATR) sampling accessory, and are reported in wave numbers (cm^-1^). Nuclear magnetic resonance spectra were recorded at 20 °C in D_2_O, CD_3_OD, or CDCl_3_ using either a Varian Unity-INOVA operating at 300 MHz or a Varian Unity operating at 500 MHz. Chemical shifts are given in ppm (δ) relative to tetramethylsilane. NMR peak assignments were made using COSY, HSQC and HMBC experiments.

*Methyl 2-deoxy-α/β-D-riboside* (**12**). To a solution of 2-deoxy-D-ribose (**11**, 200 mg, 1.5 mmol) in MeOH (3.6 mL), AcCl (100 μL) was added and the reaction was stirred at room temperature for 15 min. The reaction was neutralised by the addition of Dowex (OH^-^), filtered and concentrated. The resulting oil was purified by gradient flash ch romatography (EtOAc → MeOH/EtOAc, 1/9, v/v) to give methyl furanoside **12** (203 mg, 1.37 mmol, 91%). R*_f_* = 0.7 (15% MeOH in EtOAc); [α]_D_^18^ = +5.20 (c = 1.0, EtOH); IR (film), 3372, 2942, 1448, 1358, 1031, 1009 cm^-1^; ^1^H-NMR (500 MHz, CDCl_3_) δ 5.17 (dd, *J*_1,2b_ = 2.1, *J*_1,2a_ = 5.5 Hz, 1H, H-1β), 5.12 (dd, *J*_1,2b_ = 2.3, *J*_1,2a_ = 4.4 Hz 1H, H-1α), 4.55 (br s, 1H, H-3β), 4.16 (m, 2H, H-3α, H-4α), 4.09 (m, 1H, H-4β), 3.78-3.59 (m, 4H, H-5α, H-5β), 3.41 (s, 3H, OMe-α), 3.40 (s, 3H, OMe-β), 2.32 (ddd, *J*_1,2a_ = 2.3, *J*_2a,3_ = 4.9, *J*_2a,2b_ = 14.0 Hz, 1H, H-2aα), 2.18-2.09 (m, 2H, H-2bα, H-2aβ), 2.05-2.02 (m, 1H, H-2bβ); ^13^C-NMR (125 MHz, CDCl_3_) δ 105.7 (C1-β), 105.6 (C1-α), 87.8 (C4-β), 87.6 (C4-α), 73.0 (C3-β), 72.3 (C3-α), 63.6 (C5-β), 63.3 (C5-α), 55.6 (OMe-β), 55.0 (OMe-α), 42.8 (C2-β), 41.7 (C2-α); HRMS(ESI) *m/z* calcd. for [C_6_H_12_O_4_Na]^+^:171.0633, obsd.:171.0631.

*Methyl 2,5-dideoxy-5-iodo-α/β-D-riboside* (**10**). To a solution of methyl 2-deoxy-α/β-D-riboside (**12**, 200 mg, 1.3 mmol) in dry THF (6.5 mL) under an atmosphere of nitrogen, PPh_3_ (524 mg, 2.0 mmol), imidazole (177 mg, 2.6 mmol) and I_2_ (504 mg, 2.0 mmol) were added. The reaction was stirred at room temperature for 18 hours then filtered and concentrated. The residue was purified by gradient flash chromatography (EtOAc → MeOH/EtOAc, 1/10, v/v) to give the pure methyl 2,5-dideoxy-5-iodo-D-riboside **10**. (257 mg, 0.99 mmol, 73%). R*_f_* = 0.6 (15% MeOH in EtOAc); [α]_D_^17^ = -2.05 (c = 1.0, CHCl_3_); IR (film), 3475, 2986, 2874, 2829, 1416, 1441, 1062, 1016 cm^-1^; ^1^H-NMR (500 MHz, CDCl_3_) δ 5.14 (d, *J*_1,2b_ = 4.5 Hz, 1H, H-1β), 5.12 (dd, *J*_1,2b_ = 1.6, *J*_1,2a_ = 5.2 Hz 1H, H-1α), 4.49 (ddd, *J*_2a,3_ = 4.1, *J*_2b,3_ = 6.6, *J*_3,4_ = 10.7 Hz, 1H, H-3β), 4.14 (m, 3H, H-3α, H-4β, H-4α), 3.39 (s, 3H, OMe-β), 3.36 (s, 3H, OMe-α), 3.32 (dd, *J*_4,5a _= 5.8, *J*_5a,5b_ = 9.5 Hz, 1H, H-5aβ), 3.26 (dd, *J*_5a,4_ = 4.9, *J*_5a,5b_ = 10.5 Hz, 1H, H-5aα), 3.20 (t, *J*_5a,5b _= *J*_4,5b_ = 9.5 Hz, 1H, H-5bβ), 3.18 (dd, *J*_4,5b_ = 6.3, *J*_5a,5b_ = 10.5 Hz, 1H, H-5bα), 2.32 (ddd, *J*_1,2b_ = 1.6, *J*_2b,3_ = 6.8, *J*_2a,2b_ = 13.5 Hz, 1H, H-2aα), 2.23 (ddd, *J*_1,2b_ = 4.5, *J*_2b,3_ = 6.6, *J*_2a,2b_ = 14.2 Hz, 1H, H-2bβ), 2.13 (td, *J*_1,2a_ = *J*_2a,3_ = 5.2, *J*_2a,2b_ = 13.5 Hz, 1H, H-2bα), 2.00 (dd, *J*_2a,3_ = 4.1, *J*_2a,2b_ = 14.2 Hz, 1H, H-2aβ); ^13^C-NMR (125 MHz, CDCl_3_) δ 105.8 (C1-β), 105.4 (C1-α), 86.2 (C4-β), 86.0 (C4-α), 75.8 (C3-β), 75.6 (C3-α), 55.3 (OMe-β), 55.1 (OMe-α), 41.9 (C2-β), 40.9 (C2-α), 7.9 (C5-β), 6.7 (C5-α); HRMS(ESI) *m/z* calcd. for [C_6_H_11_O_3_INa]^+^:280.9651, obsd.:280.9655.

*(S)-5-Amino-pent-1-en-3-ol hydrochloride* (**9**). To a solution of methyl 2,5-dideoxy-5-iodo-α/β-D-riboside (**10**, 100 mg, 0.38 mmol) in a saturated solution of NH_4_OAc in EtOH (7.6 mL) was added activated Zn (124 mg, 1.9 mmol), NaCNBH_3_ (48 mg, 0.76 mmol) and 30% aqueous NH_3_ (3 mL). The mixture was stirred at reflux for 18 h, cooled to room temperature and concentrated under reduced pressure. The residue was redissolved in *i*PrOH and concentrated. The suspension was purified directly using Dowex (H^+^). The product was eluted in 5 to 15% aqueous NH_3_, and the free base was converted into the HCl salt (HCl in isopropanol) to give pure alkenylamine hydrochloride **9** (42 mg, 0.31 mmol, 81%). R*_f_* = 0.4 (DCM/EtOH/MeOH/30% aqueous NH_3_, 5/2/2/1, v/v/v/v); [α]_D_^17^ = -3.2 (c = 0.1, EtOH); IR (film), 3359, 3047, 2955, 2927, 2854, 1635, 1428, 1134, 1056 cm^-1^; ^1^H-NMR (300 MHz, D_2_O) δ 5.35 (ddd, *J*_3,4_ = 6.1, *J*_4,5b _=10.5, *J*_4,5a_ = 17.3 Hz, 1H, H4), 5.06 (dd, *J*_5a,5b _= 1.3, *J*_4,5a_ = 17.3 Hz, 1H, H-5a), 5.04 (dd, *J*_5a,5b _= 1.3, *J*_4,5b_ = 10.5 Hz, 1H, H-5b), 4.04 (q, *J*_2a,3_ = *J*_2b,3_ = *J*_3,4 _= 6.1 Hz, 1H, H-3), 2.96 (m, 2H, H-1), 1.38 (m, 2H, H-2); ^13^C-NMR (75 MHz, D_2_O) δ 138.8 (C4), 115.8 (C5), 70.1 (C3), 36.3 (C1), 32.9 (C2). HRMS(ESI) *m/z* calcd. for [C_5_H_12_NO]^+^:102.0919, obsd.:102.0921.

*(7S,7aS)-7-Hydroxy-tetrahydro-pyrrolo[1,2-c]oxazol-3-one* (**8**). To a solution of linear alkenylamine hydrochloride **9** (30 mg, 0.21 mmol) in water (2 mL) was added NaHCO_3 _(27 mg, 0.33 mmol) and I_2_ (61 mg, 0.24 mmol). The solution was stirred 18 h at room temperature then filtered and concentrated under reduced pressure. The residue was purified by gradient flash chromatography (EtOAc → EtOAc/MeOH, 99/1, v/v) to give carbamate **8** as an amorphous white powder (27 mg, 0.19 mmol, 90%). [α]_D_^17^ = +7.0 (*c* = 0.1, EtOH); IR (film), 3419, 3385, 3047, 2986, 2931, 1730, 1448, 1087 cm^-1^; ^1^H-NMR (500 MHz, D_2_O) δ 4.42 (t, *J*_4,5a_ = *J*_5a,5b_ = 9.2 Hz, 1H, H-5a), 4.34 (dd, *J*_4,5b _= 3.6, *J*_5a,5b_ = 9.2 Hz, 1H, H-5b), 4.08 (t, *J*_3,4 _= *J*_2a,3_ = 3.6 Hz, 1H, H-3), 3.90 (td, *J*_3,4 _=*J*_4,5b _= 3.6, *J*_4,5a_ = 9.2 Hz, 1H, H-4), 3.37 (ddd, *J*_1a,2b_ = 8.1, *J*_1a,2a_ = 9.5, *J*_1a,1b_ = 10.9 Hz, 1H, H-1a), 3.15 (ddd, *J*_1b,2b_ = 2.2, *J*_1b,2a_ = 10.0, *J*_1a,1b_ = 10.9 Hz, 1H, H-1b), 2.06 (ddd, *J*_1a,2a_ = 9.5, *J*_1b,2a_ = 10.0, *J*_2a,2b_ = 14.2 Hz, 1H, H-2a), 1.93 (ddd, *J*_1b,2b_ = 2.2, *J*_1a,2b_ = 8.1, *J*_2a,2b_ = 14.2 Hz, 1H, H-2b); ^13^C-NMR (125 MHz, D_2_O) δ 171.0 (C6), 69.6 (C3), 64.4 (C4), 64.3 (C5), 43.0 (C1), 33.7 (C2). HRMS(ESI) *m/z* calcd. for [C_6_H_9_NO_3_Na]^+^:166.0486 obsd.:166.0480.

*(2R,3R)-2-(Hydroxymethyl)-3-hydroxypyrrolidine hydrochloride* (**7**). To a solution of carbamate **8 **(10 mg, 0.07 mmol) in absolute EtOH (2 mL) was added NaOH (28 mg, 10 mmol). The solution was stirred at reflux for 2 h then cooled and neutralised with Dowex (H^+^). Pyrrolidine **7 **was eluted in 5 to 15% aqueous NH_3_ (8.2 mg, 0.07 mmol, 99%). The free base was converted into the HCl salt using HCl in isopropanol to give pure hydrochloride. R*_f_* = 0.1 (DCM/EtOH/MeOH/30% aqueous NH_3_, 5/2/2/1, v/v/v/v); [α]_D_^17^ = +10.0 (c = 0.09, MeOH); IR (film), 3397, 3364, 2960, 2932, 2874, 1720, 1601, 1168, 1064 cm^-1^; ^1^H-NMR (500 MHz, D_2_O) δ 4.27 (dt, *J*_2b,3_ = 1.5, *J*_2a,3_ = *J*_3,4_ = 4.9 Hz, 1H, H3), 3.98 (dd, *J*_4,5a _= 4.9, *J*_5a,5b_ = 12.1 Hz, 1H, H-5a), 3.83 (dd, *J*_4,5b _= 8.5, *J*_5a,5b_ = 12.1 Hz, 1H, H-5b), 3.31 (td, *J*_3,4 _= *J*_4,5a_ = 4.9, *J*_4,5b_ = 8.5 Hz, 1H, H-4), 3.25 (ddd, *J*_1a,2b _= 7.6, *J*_1a,2a _= 9.6, *J*_1a,1b_ = 11.6 Hz, 1H, H-1a), 3.21 (ddd, *J*_1b,2b_ = 3.4, *J*_1b,2a_ = 9.9, *J*_1a,1b_ = 11.6 Hz, 1H, H-1b), 2.13 (dddd, *J*_2a,3_ = 4.9, *J*_2a,1a_ = 9.6, *J*_2a,1b_ = 9.9, *J*_2a,2b_ = 14.1 Hz, 1H, H-2a), 2.05 (dddd, *J*_2b,3_ = 1.5, *J*_2b,1b_ = 3.4, *J*_2b,1a_ = 7.6, *J*_2a,2b_ = 14.1 Hz, 1H, H-2b); ^13^C-NMR (125 MHz, D_2_O) δ 70.8 (C3), 64.1 (C4), 59.0 (C5), 42.9 (C2), 33.3 (C1). HRMS(ESI) *m/z* calcd. for [C_5_H_12_NO_2_]^+^:118.0868 obsd.:118.0870.

## Conclusions

In summary, we have achieved an efficient, high yielding and diastereoselective protecting-group-free synthesis of 1,2,4-trideoxy-1,4-imino-L-xylitol. During the course of this work, we were able to illustrate the extension of our Vasella-reductive amination and carbamate annulation methodologies to the synthesis of 2-deoxy aza-sugars. Little is known about the biological profile of 1,2,4-trideoxy-1,4-imino-L-xylitol and this is currently under investigation.
